# 

**Published:** 1911-01

**Authors:** 


### BOOKS RECEIVED

Modern Treatment; The Management of Disease with Medicinal and Non-Medicinal Remedies. By eminent American and English authorities. Edited by Hobart Amory Hare, M.D., Professor of Therapeutics and Materia Medica, Jefferson Medical College, Philadelphia. Author of "A Textbook of Practical Therapeutics," "A Textbook of the Practice of Medicine," etc. In two very handsome octavo volumes, comprising 1,800 pages, with numerous engravings and full-page plates. Lea & Febiger, Publishers, Philadelphia and New York, 1910. (Price, per volume in cloth, $6.00; half morocco, $7.50, net.)

Anemia. By Dr. P. Erlich, Frankfort-on-Main, and Dr. A. Lazarus, Berlin. Part I, Vol. 1, Normal and Pathological Histology of the Blood. Second German edition. Translated by H. W. Armit, M.R.C.S., L.R.C.P., London. Octavo pp. 230. Illustrated. New York: Rebman Co. (Cloth, $4.00.)

Progressive Medicine, Vol. 12, December, 1910. A quarterly Digest of Advances, Discoveries and Improvements in the Medical and Surgical Sciences. Edited by Hobart Amory Hare, M.D., Professor of Therapeutics and Materia Medica in the Jefferson Medical College of Philadelphia, and Leighton F. Appleman, instructor in therapeutics at the same college. Philadelphia and New York: Lea & Febiger. (Per annum, paper bound, $6.00; cloth, $9.00.)

Hints for the General Practitioner in Rhinology and Laryngology. By Dr. Johann Fein, Vienna. Translated by J. Bowring Horgan, M.B., B. Ch., London. 12 mo, pp. 239. Illustrated with 40 figures and 2 photographic plates. New York: Rebman Co. (Cloth, $1.50.)

Evolution and Heredity. By David Berry Hart, M.D., F.R.C.P.E., Lecturer on Midwifery and Diseases of Women, School of the Royal Colleges, Edinburgh. 12 mo, pp. 259. Illustrated. New York: Rebman Co. (Cloth, $2.00.)

Mental Symptoms of Brain Disease. By Bernard Hollander, M.D. With preface by Dr. Jul. Morel, late Belgian State Commissioner in Lunacy. 12 mo, pp. 255. New York: Rebman Co. (Cloth, $2.00.)

Modern Treatment of Alcoholism and Drug Narcotism. By C. A. McBride, M.D., London. 12 mo, pp. 384. New York: Rebman Co. (Cloth, $2.00.)

Lessons on the Eye. By Frank L. Henderson, M.D., Ophthalmic Surgeon to St. Mary's Infirmary. Fourth edition. 12 mo, pp. 242. Illustrated. Philadelphia: P. Blakiston's Son & Co. 1910. (Cloth, $1.50.)

Prevention of Sexual Diseases. By Victor G. Vecki, M.D., San Francisco. With introduction by William J. Robinson, M.D. 12 mo, pp. 132. New York: Critic and Guide Co. (Cloth, $1.50.)

Proceedings of the National Confederation of State Medical Examining and Licensing Boards. Twentieth annual convention held at St. Louis, June 6, 1910. Edited by Murray Galt Motter, M.D.

Non-surgical Treatment of Duodenal Ulcer. By George Herschell, M.D., London.

The Practical Medicine Series. Ten volumes. Under the general editorial charge of Gustavus P. Head, M.D. Vol. VIII Therapeutics, Preventive Medicine, Climatology. Edited by George F. Butler, M.D., Henry B. Farill, M.D., and Norman Bridge, M.D. Series 1910. Chicago: The Year Book Publishers. (Price, $1.50; entire series, $10.00.)

The Treatment of Disease. A Manual of Practical Medicine. By Reynold Webb Wilcox, M.A., M.D., Professor of Medicine (retired), at the New York Post-Graduate Medical School and Hospital. Third edition, thoroughly revised and enlarged. Octavo, pp. 1048. Philadelphia: P. Blakiston's Son & Co. 1911. (Cloth, $7.50.)

Practical Bacteriology, Blood Work and Animal Parasitology. By E. R. Stitt, A.B., Ph.G., M.D., Surgeon U. S. Navy. Second edition, revised and enlarged. With 91 illustrations. 12 mo, pp. 345. Philadelphia: P. Blakiston's Son & Co. 1910. (Cloth, $1.50.)

Care of the Patient. A Book for Nurses. By Alfred T. Hawes, A.M., M.D. 12 mo, pp. 173. With 6 illustrations. Philadelphia: P. Blakiston's Son & Co. 1911. (Cloth, $1.00, net.)

International Clinics. A Quarterly of Illustrated Clinical Lectures and especially prepared articles on Treatment, Medicine, Surgery, Neurology, Pediatrics, Obstetrics, Gynecology, Orthopedics, Pathology, Dermatology, Ophthalmology, Otology, Rhinology, Laryngology, Hygiene, etc. Edited by Henry W. Cattell, M.D. Vol. IV, twentieth series. Philadelphia and London: J. B. Lippincott Co. 1910. (Cloth, $2.00.)

